# Comparison of a metronome-guided prehospital medication infusion technique with standard calculation: a simulated randomized, controlled, cross-over study

**DOI:** 10.1186/s12873-021-00503-6

**Published:** 2021-10-07

**Authors:** Samuel M. Galvagno, James Cloepin, Jeannie Hannas, Kurt S. Rubach, Andrew Naumann, Jonathan Wendell

**Affiliations:** 1 Department of Anesthesiology, Program in Trauma, University of Maryland School of Medicine, 22 S Greene Street, T5S18, Baltimore, MD 21201 USA; 2 Anne Arundel County Fire Department, Millersville, MD 21108 USA; 3 Baltimore-Washington Medical Center, Glen Burnie, Baltimore, MD 21061 USA; 4 Maryland Institute for Emergency Medical Services Systems (MIEMSS), 653 West Pratt Street, Baltimore, MD 21201 USA; 5 Department of Emergency Medicine, University of Maryland Baltimore Washington Medical Center, Glen Burnie, Baltimore, MD 21061 USA

**Keywords:** Prehospital, Emergency medical services, Medication infusion, Medical calculation, Medication dosing, Resuscitation

## Abstract

**Background:**

Limited research regarding administration of timed medication infusions in the prehospital environment has identified wide variability with accuracy, timing, and overall feasibility. This study was a quality improvement project that utilized a randomized, controlled, crossover study design to compare two different educational techniques for medication infusion administration. We hypothesized that the use of a metronome-based technique would decrease medication dosage errors and reduce time to administration for intravenous medication infusions.

**Methods:**

Forty-two nationally registered paramedics were randomized to either a metronome-based technique versus a standard stopwatch-based technique. Each subject served as a control. Subjects were asked to establish an infusion of amiodarone at a dose of 150 mg administered over 10 min, simulating treatment of a hemodynamically stable patient with sustained monomorphic ventricular tachycardia. Descriptive statistics and a repeated measures mixed linear regression model were used for data analysis.

**Results:**

When compared to a standard stopwatch-based technique, a metronome-based technique was associated with faster time to goal (median 34 s [IQR, 22–54] vs 50 s; [IQR 38–61 s], *P* = 0.006) and fewer mid-infusion adjustments. Ease of use was reported to be significantly higher for the metronome group (median ranking 5, IQR 4–5) compared to the standard group (median ranking 2, IQR 2–3; *P* < 0.001).

**Conclusions:**

Knowledge regarding a metronome technique may help EMS clinicians provide safe and effective IV infusions. Such a technique may be beneficial for learners and educators alike.

## Introduction

Delivery of intravenous (IV) infusions of fluid and medications is a challenging but foundational skill for prehospital clinicians [1]. An infusion pump is preferred to ensure appropriate dosing, but such devices are expensive, require extensive training, and are not available in many EMS systems. Even when using intravenous infusion flow regulators, deviations from expected infusion volumes are common and potentially harmful [2, 3]. Conventional calculation of prehospital IV infusion rates requires a mathematical computation based on the medication dose, delivery interval, and size of the IV administration set. Thus, a method to mitigate the difficulty and potential for error when administering prehospital IV infusions could assist EMS clinicians by facilitating accurate infusion control. Knowledge regarding techniques that might help EMS clinicians provide safe and effective IV infusions would be beneficial for learners and educators alike.

As the scope of prehospital medicine continues to evolve, the ability to quickly and safely administer IV medication infusions will remain an essential skill to enhance patient outcomes. For example, in patients with stable ventricular tachycardia, early administration of amiodarone has been associated with improved outcomes and following a bolus out-of-hospital cardiac arrest (OHCA) patients, an infusion is recommended [4, 5].

The objective of this study was to assess the effectiveness, accuracy, and ease of use of a metronome-guided IV medication infusion technique. We hypothesized that when compared to conventional prehospital IV medication infusion techniques, the metronome technique would be associated with fewer errors and decreased time to target infusion rate.

## Methods

Nationally registered paramedics (NRP) from Anne Arundel County Fire Department, Maryland, USA, served as subjects during this study. Subjects were randomly selected from 42 paramedic stations in the county. No more than 4 subjects from any single station were included. This study was deemed exempt from institutional review board oversight (HP-00096185) at the University of Maryland School of Medicine since this project did not involve actual human patients and was a quality improvement project designed to assess the effectiveness of an educational technique. The study was conducted in a stationary ambulance, where participants were asked to correctly establish an intravenous infusion rate using one of two techniques. The study was a conventional two-sequence, two-period, two-intervention crossover trial (i.e., 2 × 2 or AB/BA design) [6]. A permuted-block randomization scheme was generated using the -*egen* suite of command sequences available in Stata/SE Version 15.1 (Stata Corp, College Station, TX). This scheme determined whether each subject would begin with either a “standard calculation” using microdrippers and a calculation or a “metronome” technique using an audible cadence to establish the correct medication infusion rate.

Each subject served as her or his own control; the order of medication infusion rate performance was reversed after the initial assigned technique was complete. Primary outcomes of interest included time to target infusion rate (from start to finish), of use time to goal medication infusion rate, and total elapsed time. Secondary outcomes included the number of adjustments required to maintain a stable infusion rate. Ease of use and demographic data were evaluated with a 5-point Likert scale with a value of “1” indicating most difficult and “5” indicating easiest, via a written survey that was given to each participate at the conclusion of the study.

Subjects were asked to establish an infusion of amiodarone at a dose of 150 mg administered over 10 min, simulating treatment of a hemodynamically stable patient with sustained monomorphic ventricular tachycardia. Multiple IV administration sets (15-, 20-, and 60 drips/mL) were used to simulate restocking from different receiving hospitals; all three sets were available to each subject.

The amiodarone vial (3 mL) was injected into a 100 mL IV bag of 5% dextrose in water. Subjects were required to use the following formula to establish the correct infusion rate (drops):

$$ \mathbf{Volume}\ \left(\mathbf{mL}\right)/\mathbf{Minutes}\ \mathbf{x}\ \mathbf{Infusion}\ \mathbf{Set}\ \mathbf{Drip}\ \mathbf{Factor}\ \left(\mathbf{drops}/\mathbf{mL}\right)=\mathbf{Flow}\ \mathbf{Rate}\ \left(\mathbf{drops}/\mathbf{\min}\right) $$

Formula 1. Intravenous infusion set calculation.

For example, using a volume of 103 mL of amiodarone, at 20 drops/mL, the flow rate for a 10-min infusion was 206 drops/min. Once the flow rate was calculated, subjects were instructed to establish an infusion of amiodarone using either the standard or metronome technique according to the randomization scheme. For the standard technique, a watch was used to synchronize the drops/min. In the metronome group, a Quik Time® metronome (John Hornby Skews & Co., Leeds, UK) was used. Depending on the drips/min per the infusion set used, the metronome cadence was adjusted to match the desired flow rate.

Using a one-way ANOVA to detect a statistically significant difference of 2 min between techniques (time to infusion goal rate) with a standard deviation of 1 min, at an alpha of 5% and with 80% power, a total of 12 patients were required. Post hoc, additional sample size calculations were performed after we observed significantly shorter time-to-target infusion goals in both groups than we hypothesized. For a two-sample paired t-test; a total of 34 participants were required in each group to detect a 1-min improvement in performance time at 80% power, an alpha of 5%, and with a standard deviation of 30 s.

Descriptive statistics were used to describe the data. The Shapiro-Wilk test and q-q plots were used to assess normality of the data, followed by application of the appropriate parametric or nonparametric statistical test. Multiple linear regression was used to adjust for potential known confounders. With a dependent variable of mean time-to-target infusion goal, a repeated measures mixed linear regression model was constructed. Independent variables were added, to include years of experience as a paramedic, work as an interfacility critical care transport medic, and additional training. Ease of use (Likert scale) data were analyzed using the Wilcoxon rank sum test due to the nonparametric distribution of the data. All tests were two-tailed and a *P* value of < 0.005 was considered statistically significant [7]. Analyses were performed in Stata/SE version 15.1 (Stata Corp, College Station, TX) and GraphPad Prism 7.0d (GraphPad Software, La Jolla, CA).

## Results

**Fig. 1:**
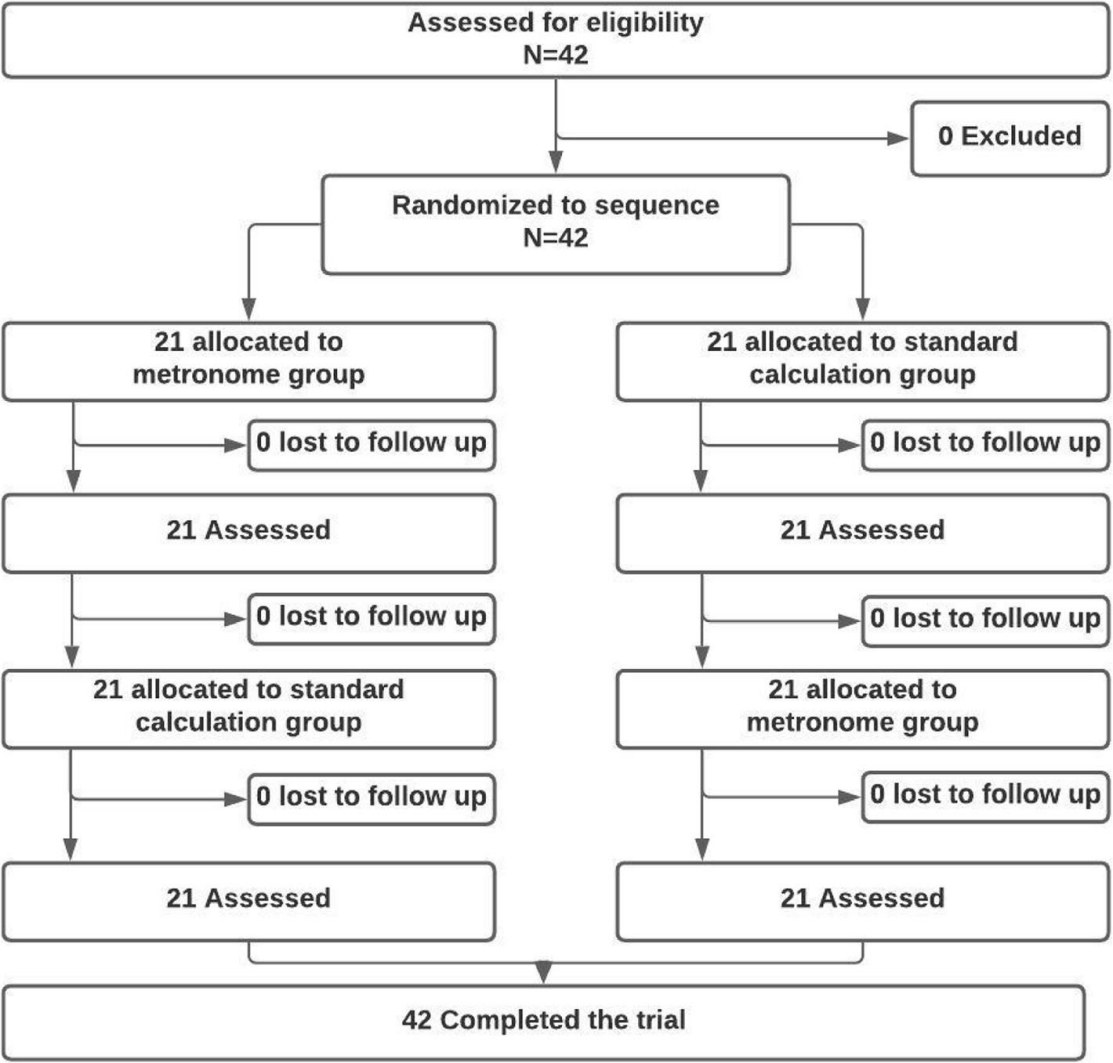
CONSORT study flow diagram

Forty-two NRPs were enrolled (Fig. 1).

The mean number of years each subject had worked as an NRP was 10.4 (6.7). Twelve (14.6%) NRPs had prior experience working for critical care transport organizations, none were registered nurses, and two (2.4%) were physician’s assistants.

The 15 drips/mL set was used by 15% of all subjects and the 20 drips/mL set was used by 85%; no subjects chose the 60 drips/mL set. In the standard group, adjustments to the drip rate were required 63% of the time; in the metronome groups, adjustments were required in 34.4% (*P* = 0.14). Infusions were finished prematurely 9.8% of the time in the standard group vs. 4.9% of the time in the metronome group (*P* = 0.29).

**Fig. 2:**
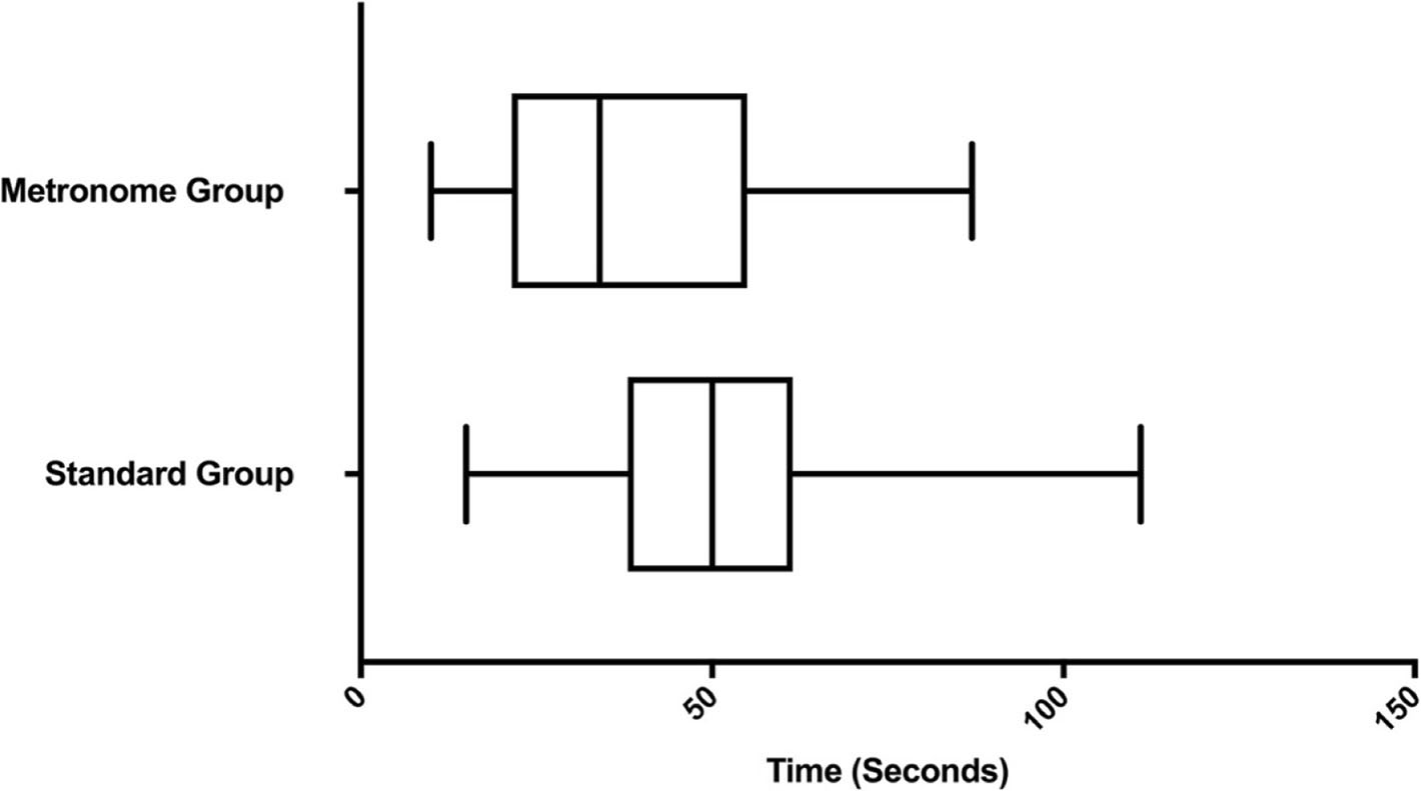
Box and whisker plots depicting median time to infusion rate goal. *P* = 0.006

Time to infusion completion was not significantly different between the groups (standard group, mean time 460.9 s [118.7 s] vs. metronome group, mean time 510.8 s [116 s]; *P* = 0.19). Median time to goal was significantly faster in the metronome group (median 34 s; IQR, 22–54 s) compared to the standard group (median 50 s; IQR 38–61 s) (*P* = 0.006) (Fig. 2).

**Fig. 3:**
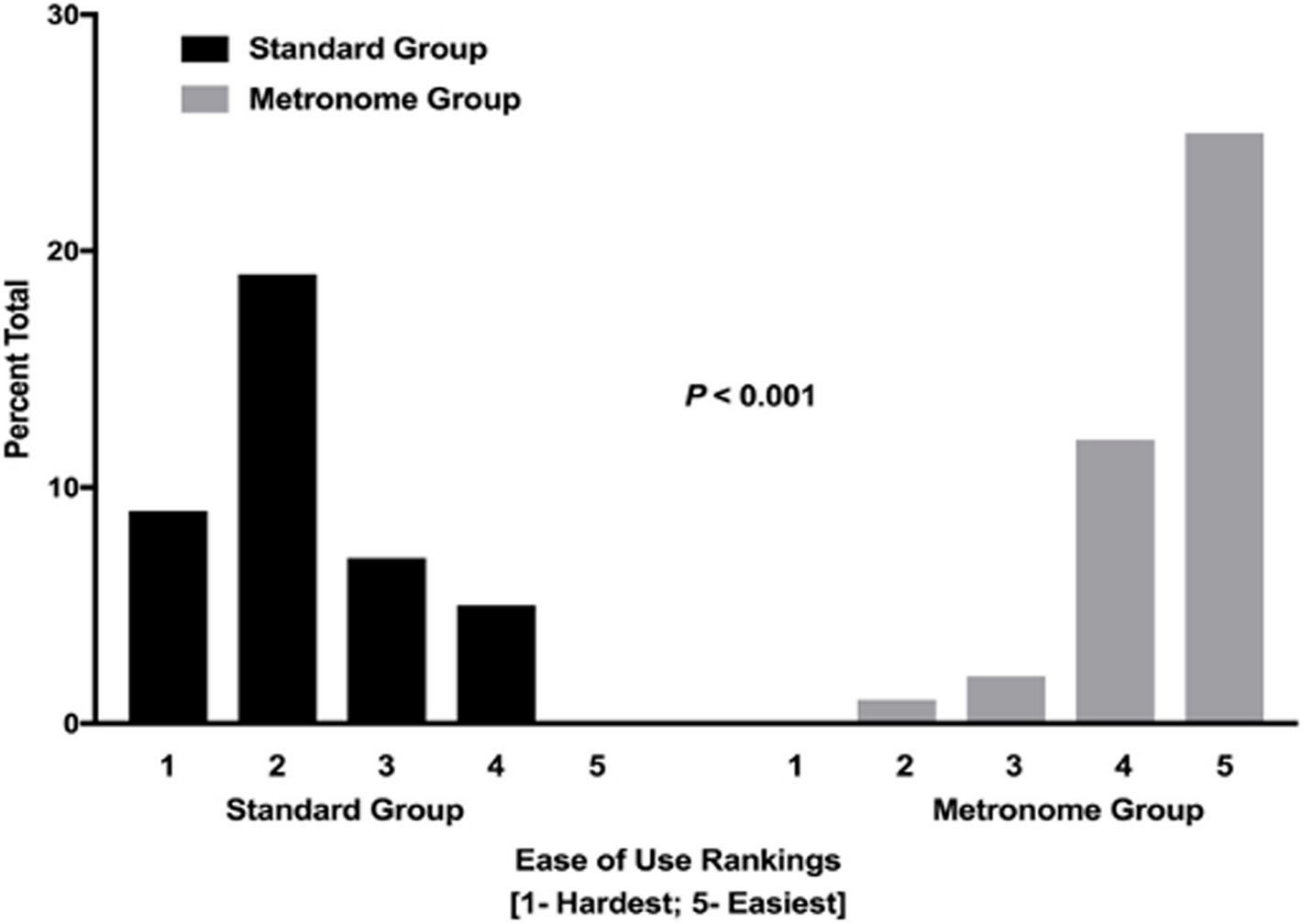
Self-reported ease of use

When controlling for total years of experience and critical care transport experience with multiple linear regression, time to goal infusion rate was significantly faster in the metronome group (mean of 13 s faster; 95% confidence interval, 22–7 s; *P* < 0.001). We assessed our regression model for linearity of the relationships between covariates and the outcome of interest, homogeneity of the residuals, absence of measurement error in the predictor, and distribution of random effect coefficients. All assumptions were satisfied. Ease of use was reported to be significantly higher for the metronome group (median ranking 5, IQR 4–5) compared to the standard group (median ranking 2, IQR 2–3; *P* < 0.001) (Fig. 3).

## Discussion

Administration of intravenous medication infusions in the prehospital arena is an arduous task. Even when using intravenous infusion flow regulators, significant deviations from volume and flow rates are possible, leading to potential medical delivery errors [2]. When delivering fluid volumes alone, accuracy may be highly variable [8]. In this randomized, cross over study, use of a metronome was associated with greater ease of use and shorter time to goal infusion rate.

The results of this study have significant implications for prehospital clinicians. Infusions of lidocaine and amiodarone may be associated with clinically relevant outcomes, and proper dosing is essential. In one large randomized double-blind study [9], both of these agents have been associated with improved survival to hospital admission [5], and amiodarone has been shown to be moderately effective for the treatment of sustained ventricular tachycardia [4]. Amiodarone is also associated with improved neurological outcome at hospital discharge for out-of-hospital cardiac arrest in patients who had ventricular fibrillation as the presenting dysrhythmia [10].

The prehospital literature is largely devoid of data describing safe and efficient practices for medication infusion delivery. Park et al. compared four methods of infusion control in a cohort of emergency medicine nurses, highlighting the inaccuracy of dial flow controllers and supporting the use of an “intravenous infusion therapy helper” based on a metronome and drop counter [11]. A phone application with a metronome-like flow rate control function was used control the volume of a fluid infusion. Compared to a stopwatch technique, the volume of fluid was more precise with the metronome-based technique, although an additional control was added using a control function within the application to add additional precision. The outcome of interest in this study was fluid volume, not medication dosing. In our study, use of an off-the-shelf metronome demonstrated superior effectiveness and ease of use. Metronome applications for smartphones are prevalent, free, and can be used in the field to adjust medication flow rates. Such techniques could prove useful for EMS jurisdictions administering infusions that require timing, such as amiodarone, lidocaine, magnesium, epinephrine, and others.

There are a number of limitations to our work. The crossover nature of this study may have introduced confounding due to carryover between technique assignments since the metronome technique might have helped the participants remember a correct drip cadence. Although the metronome group had a lower overall number of premature infusion completions, compared to the standard technique, this difference was not statistically significantly different, likely due to the sample size. Although randomized and administered in a simulated prehospital setting, this study did not involve actual patients and only one medication infusion was assessed. It is possible that other medication infusions may be subject to a greater or lesser degree of error. Prehospital IV infusions are not used in all EMS jurisdictions; hence, it is possible that the results of this study may not be externally generalizable. However, for jurisdictions in developing countries or austere settings, our results have implications for education and training, especially when financial constraints exist. Finally, we hypothesized that a significant difference in time to target infusion rate would be observed; we hypothesized that a far faster time to goal in the metronome group would be observed. NRPs in both groups achieved a time to goal that was significantly faster than we hypothesized (Fig. 2). Therefore, it is likely that this study was underpowered to detect a clinically meaningful difference in the primary outcome of interest—time to target infusion rate.

## Conclusion

Precise control of prehospital medication infusions is required to prevent harm and maximize therapeutic effectiveness. As many EMS jurisdictions across the world do not have access to electronic infusion pumps, novel techniques are required for training and clinical practice. In this study, the use of a metronome technique was associated with faster time to goal infusion rate and greater ease of use compared to standard techniques.

## Data Availability

The datasets generated and/or analysed during the current study are located in the METRONOMEEMS repository persistent web link to datasets: https://www.dropbox.com/s/3uz6rh4xz70p56z/metronome_data.dta?dl=0.
